# Concept for individualized patient allocation: ReCompare—remote comparison of particle and photon treatment plans

**DOI:** 10.1186/1748-717X-9-59

**Published:** 2014-02-18

**Authors:** Armin Lühr, Steffen Löck, Klaus Roth, Stephan Helmbrecht, Annika Jakobi, Jørgen B Petersen, Uwe Just, Mechthild Krause, Wolfgang Enghardt, Michael Baumann

**Affiliations:** 1 OncoRay—National Center for Radiation Research in Oncology, Medical Faculty and University Hospital Carl Gustav Carus, Technische Universität Dresden, Fetscherstr. 74, PF 41, 01307 Dresden, Germany; 2 German Cancer Consortium (DKTK), Dresden, Germany and German Cancer Research Center (DKFZ), Heidelberg, Germany; 3 Department of Radiation Oncology, University Hospital Carl Gustav Carus, Technische Universität Dresden, Fetscherstr. 74, 01307 Dresden, Germany; 4 Institute of Radiation Oncology, Helmholtz-Zentrum Dresden-Rossendorf, Dresden, Germany; 5 Department of Medical Physics, Aarhus University Hospital, Aarhus, Denmark

**Keywords:** Proton therapy, Particle therapy, Patient selection, Treatment plan comparison

## Abstract

**Background:**

Identifying those patients who have a higher chance to be cured with fewer side effects by particle beam therapy than by state-of-the-art photon therapy is essential to guarantee a fair and sufficient access to specialized radiotherapy. The individualized identification requires initiatives by particle as well as non-particle radiotherapy centers to form networks, to establish procedures for the decision process, and to implement means for the remote exchange of relevant patient information. In this work, we want to contribute a practical concept that addresses these requirements.

**Methods:**

We proposed a concept for individualized patient allocation to photon or particle beam therapy at a non-particle radiotherapy institution that bases on remote treatment plan comparison. We translated this concept into the web-based software tool ReCompare (REmote COMparison of PARticlE and photon treatment plans).

**Results:**

We substantiated the feasibility of the proposed concept by demonstrating remote exchange of treatment plans between radiotherapy institutions and the direct comparison of photon and particle treatment plans in photon treatment planning systems. ReCompare worked with several tested standard treatment planning systems, ensured patient data protection, and integrated in the clinical workflow.

**Conclusions:**

Our concept supports non-particle radiotherapy institutions with the patient-specific treatment decision on the optimal irradiation modality by providing expertise from a particle therapy center. The software tool ReCompare may help to improve and standardize this personalized treatment decision. It will be available from our website when proton therapy is operational at our facility.

## Background

A promising strategy to improve the treatment of cancer is to apply patient-specific, technologically optimized radiotherapy that may also include particle irradiation such as proton or carbon ion beam therapy. While the number of particle therapy centers worldwide increases steadily [1] the relative number of cancer patients who are treated with this technique—and may therefore benefit from its potential advantages—remains small compared to those patients treated with conventional photon therapy. To guarantee an equal and sufficient access to optimal radiotherapy, it is essential to identify those patients that have a higher chance to be cured with fewer side effects by particle therapy than by state-of-the-art photon therapy. Thus, patients need to be individually allocated to exactly that irradiation type that offers them the best chance for cure.

Currently, it is still subject of investigation on which criteria an attending physician should base a final decision regarding the treatment modality for an individual patient. One practical solution is a direct comparison of patient-specific dose distributions to discriminate between treatment options [2,3]. This may also include the application of available state-of-the-art tumor control probability and normal tissue complication probability models [4,5]. For example, in the Netherlands a two-step approach of dose comparison and complication prediction is compulsory for reimbursing model-based indications [2].

Most cancer patients will initially be referred to a non-particle radiotherapy institution, which only has access to conventional irradiation modalities and therefore has only limited experience with particle therapy. The identification of patients that benefit most from radiotherapy with particles requires initiatives by particle as well as non-particle radiotherapy centers to form networks, to establish a procedure for the decision process, and to implement means for the remote exchange of relevant patient information.

In response to these requirements our objective was twofold: we wanted to

1. propose a concept for individualized patient allocation to photon or particle radiotherapy, based on remote treatment plan exchange and comparison;

2. develop a software tool that translates this concept into practical use.

A purposeful software solution that supports the identification and allocation of patients should meet several specifications, namely, realize remote transmission of patient data between radiotherapy institutions; require minimal time and financial effort from users; comply with standard treatment planning systems; integrate into routine clinical workflow; and consider safety measures and IT requirements for the use in clinical environments including patient data protection issues.

## Methods

### Concept of individualized patient allocation to particle therapy

Currently, the available clinical and radiobiological data for particle irradiation are for most cases insufficient to exclusively base a treatment decision on them [6,7]. Instead, patient allocation that relies on patient-specific treatment plan comparison appears to be more robust [8] and is therefore the basis for our concept, which is explained in what follows.

The (initial) treatment decision for most patients will be made at a non-particle radiotherapy institution that lacks the competence of particle therapy planning. Nevertheless, radiation oncologists at non-particle radiotherapy institutions are clearly qualified to identify treatment plans that do not meet the criteria of the prescription (e.g., do not deliver the required dose to the tumor at acceptable levels of expected complications). For such cases, experienced personnel at the particle therapy center generate a particle therapy plan on the basis of the patient data accumulated at the non-particle radiotherapy institution, but with a different planning target volume (PTV) that considers the uncertainties at the particle therapy center. After receiving this particle treatment plan the non-particle radiotherapy institution performs a comparison with its own state-of-the-art photon plan and may finally come up with an individualized treatment decision whether to allocate the patient to a treatment with either photon or particle therapy. Note, this concept implies the important aspect of cooperation and exchange between a non-particle radiotherapy institution and a particle therapy center, which is an integral part of a well-founded decision on the different treatment options.

**Figure 1:**
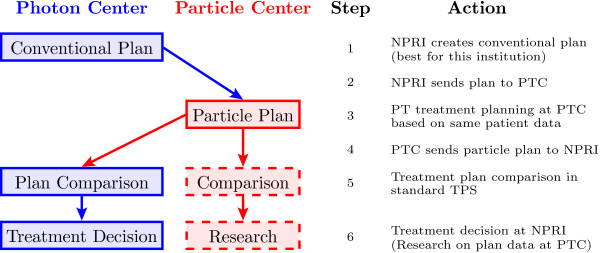
**Schematic workflow of treatment decision process based on plan comparison for photon and particle irradiation.** The software ReCompare provides functionality to exchange the plans between the photon and particle therapy centers and to store plans in a database, i.e., it enables steps 2 to 5 (cf. text for details). NPRI: non-particle radiotherapy institution; PTC: particle therapy center; TPS: treatment planning system.

The workflow (depicted schematically in Figure 1) includes the preparation and transmission of patient and treatment plan data [steps 2 to 5]. The exchange is realized by the developed software tool ReCompare (REmote COMparison of PARticlE and photon treatment plans)—cf. the manuscript (Lühr et al.: Implementation of ReCompare – remote comparison of particle and photon treatment plans, in preparation) for implementation details. The preparation of the photon or particle treatment plan as well as the (final) plan comparison are performed at each institution with the same treatment planning system that is routinely used for this purpose to integrate the exchange process into clinical workflow. Patient data acquired during the application of ReCompare, i.e., pairs of pseudonymized conventional and particle therapy plans, are available from the database. They may be subject to later internal comparison for quality assurance as well as serve as a valuable data basis for clinical research regarding different irradiation modalities. The exchange, comparison, and optional scientific analysis of patient data require the informed consent of the patient. A document for this purpose—containing information for patients on the data handling—will be provided to the external non-particle radiotherapy institutions, which considers the applicable law and regulations, in particular the (German) data protection law.

### Structure of ReCompare

**Figure 2:**
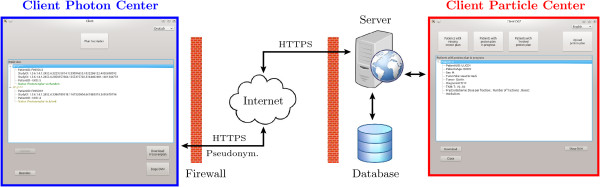
**Structure of the remote plan exchange software ReCompare.** It consists of two different clients: one for photon and one for particle therapy centers, respectively. They connect via HTTPS with the central server module, which provides a database storing the patient data for the exchange. The two implemented languages English and German are demonstrated.

The remote treatment plan exchange software ReCompare consists of three main components (as schematically depicted in Figure 2): (1) a local client at (each of) the non-particle radiotherapy institution(s), (2) one client at the particle therapy center, and (3) one central server module with a database.

The communication and data transfer between clients and the server are performed based on the Hypertext Transfer Protocol Secure (HTTPS) [9] to ensure secure data transmission. The server communicates via the internet with clients at external non-particle radiotherapy institutions and via a local network connection with the client of the particle therapy center. We preferred HTTPS to other secure protocols (e.g., File Transfer Protocol over Transport Layer Security [FTPS]) for two reasons: first, other protocols than HTTPS are not allowed by many institutions and second, HTTPS allows performing the entire communication (e.g., database requests) additionally to the data transfer through one interface. In general, individual processing steps during the plan exchange process are checked for consistency and the exchange is terminated in the case that a test fails.

A complete plan data set consists of data files in Digital Imaging and Communications in Medicine (DICOM) format [9] containing the patient computed tomography (CT), the radiation therapy (RT) structure set, the RT plan, and the RT dose distribution. Before uploading a photon plan, the files in DICOM format are (i) tested for completeness and consistency, (ii) pseudonymized, while (iii) a subset of the pseudonymized patient data (e.g., patient ID and birth date) is—after encryption using the Advanced Encryption Standard (AES) [10]—stored in a file. In contrast to fully anonymized data sets, in which all patient identifying fields are replaced, pseudonymization preserves some less identifying patient information (e.g., patient age and radiotherapy center) that might be of relevance for the treatment process. The pseudonymization has to preserve the intrinsic relations between the DICOM files, i.e., replacing the intrinsic referencing identifiers with pseudonyms in a consistent way. Otherwise, a subsequent DICOM import would fail, since the set of DICOM files could not be recognized to belong to one treatment plan. Due to the procedures in (ii) and (iii) only the non-particle radiotherapy institution can identify the patient and access patient-identifying data (e.g., name and date of birth) with their local encryption key. The checks in (i) intend to prevent faulty operation (e.g., selected patient case already exists) and to test consistency of the data (e.g., selected DICOM files do not belong together or do not form a complete plan) as well as the successful completion of processing steps (e.g., pseudonymization or writing to the database). During the initial upload process of a photon plan, the user can provide additional metadata, such as tumor location and fractionation scheme, which are then available to the particle therapy center for the purpose of treatment planning.

The final plan comparison takes place at the non-particle radiotherapy institution where the attending physician is responsible for the treatment decision. For this purpose the ReCompare software writes the dose distribution of a particle plan into a template file for a photon dose distribution in DICOM format that can be imported into photon treatment planning systems. Also during the particle plan upload, the staff at the particle therapy center may add supplementary notes regarding, e.g., the robustness of the particle plan, that are of relevance for a treatment decision. After the download of the particle plan, which is based on the same CT as the photon plan, the comparison of the dose distributions takes place in the standard treatment planning system of the non-particle radiotherapy institution and according to the procedure of that institution, which may base, e.g., on a dose volume histogram and a slice-by-slice dose level evaluation.

ReCompare fits seamlessly in the daily clinical workflow by being compatible with a number of standard treatment planning systems. These systems support planning for photon and/or proton irradiation while compatibility with planning systems capable of ions heavier than protons is under development. The configuration of the client software is kept simple and requires minimal effort by all users. The installation of the server part has to be performed in accordance to relevant requirements for protecting patient data in that country. As an example, the design of the server installation at our facility is discussed in the manuscript (Lühr et al.: Implementation of ReCompare – remote comparison of particle and photon treatment plans, in preparation).

## Results

We developed and tested the software tool ReCompare (REmote COMparison of PARticlE and photon treatment plans), which facilitates the exchange of photon and particle treatment plans between different radiotherapy institutions.

**Figure 3:**
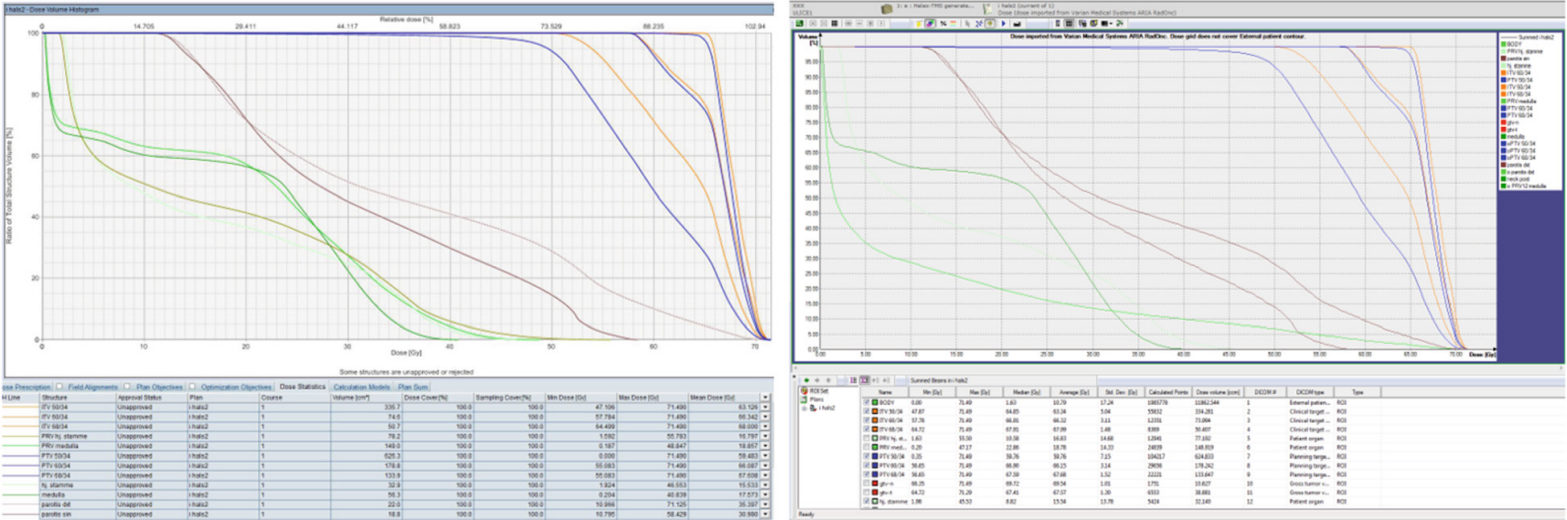
**Remote transmission of photon treatment plan of a head and neck cancer patient.** Dose volume histograms. Left: the plan was created in Aarhus, Denmark using Eclipse. Then ReCompare pseudonymized the plan and sent it via HTTPS to Dresden, Germany. Right: after download from the server the plan was visualized in Dresden with Oncentra Masterplan.

The web-based approach of ReCompare allows true remote transmissions of patient data and plans, in principle, between any two radiotherapy centers that have access to the internet. This includes the plan exchange also between different countries as shown in Figure 3 for the example of a photon plan for a head and neck cancer patient that was transmitted between the university hospitals Aarhus, Denmark and Dresden, Germany. The comparison of the dose volume histograms shows that the file transfer between different treatment planning systems (here between Eclipse and Oncentra Masterplan) via ReCompare preserves the quantitative dose information.

**Figure 4:**
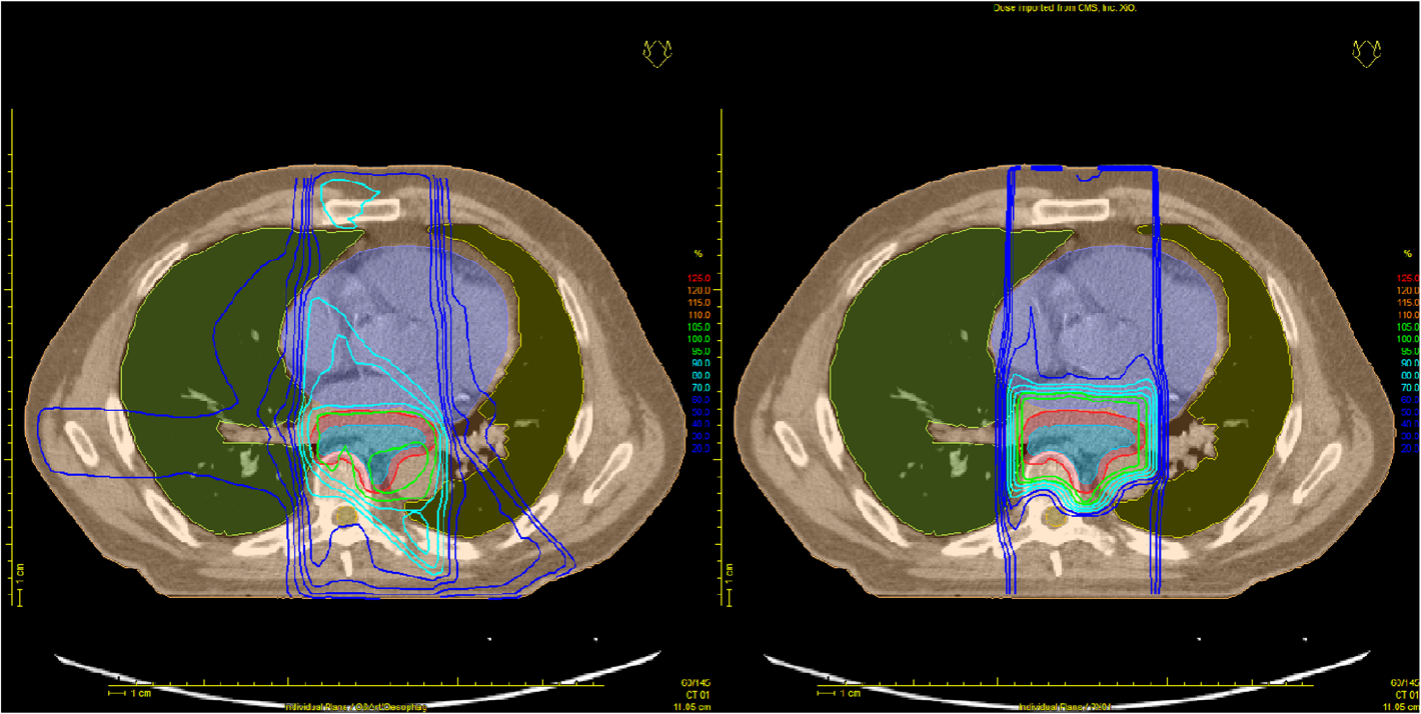
**Comparison of photon (left) and proton (right) treatment plans for the same esophagus cancer patient.** The plan exchange was performed with ReCompare. The proton plan was planned with XiO and the photon plan with Oncentra Masterplan, which was also used for the displayed plan comparison.

The usability of the concept and its implementation was confirmed by clinical personnel for various body sites, including lung, head and neck, prostate, and breast. Clinically, these test applications showed that potentially relevant advantages of one irradiation modality over the other appear to be patient specific and cannot easily be generalized. Figure 4 shows the direct comparison of a photon with a proton treatment plan as done at a non-particle radiotherapy institution for an esophagus cancer patient.

ReCompare is—due to its design—easy to use and requires only minimal effort by the user. The focused functionality and the small number of buttons in the graphical user interface of the local clients ensure easy usability and reduce the chance of unintended faulty operations. The latter are also prevented by automatic consistency checks before and after individual processing steps considering, e.g., completeness of treatment plans and success of the upload process. It takes about 1 minute to initiate (selecting the matching DICOM folder and providing metadata) an upload of a photon plan to the server. The following transfer of all files of a plan via the internet took in our tests also approximately 1 minute (total file size about 100 MB). The duration depends, however, on the total file size of the plan and the speed of the internet connection. The local client comes with a free to use license and its setup is kept simple. The user can choose between different languages: currently English and German are supported.

## Discussion

We proposed a concept that enables non-particle radiotherapy institutions to actively participate in allocating their patients to either state-of-the-art photon or particle therapy based on a patient-specific treatment plan comparison for cases when the optimal photon plan fails to fulfill the prescription criteria. Furthermore, we translated this concept into the software tool ReCompare (REmote COMparison of PARticlE and photon treatment plans), which realizes web-based remote transmission of patient data and thereby allows to compare conventional with particle treatment plans at a non-particle radiotherapy institution. ReCompare is easy to use, freely available, protects patient data, and is compatible with several standard treatment planning systems. It has therefore the potential to seamlessly integrate into clinical work flow.

The treatment decision where to allocate the patient is also based on other relevant factors (beyond the comparison of dose distributions), such as age, tumor location or radioresistance. Recommendations without the need for plan comparison—and therefore without ReCompare—can be given for indications where particle irradiation results in a clear advantage or disadvantage. Examples are diagnoses that are either accepted indications for particle treatment (e.g., following international guidelines) or indications for which no particle treatment is established, respectively. We expect, however, that momentarily most cases do not belong to one of these groups. Then, dose comparison is the only robust method to quantify differences and potential benefits between the treatment modalities for an individual patient [7]. They are therefore requested by many authorities before they approve coverage of treatment expenses [2,11]. The Radiation Oncology Collaborative Comparison (ROCOCO) consortium performs multicentric *in silico* trials comparing photon and particle radiotherapy on the level of treatment plan comparison. Their results for non-small cell lung cancer indicate that the best treatment modality should be investigated on an individual patient basis [8] and are therefore in line with our findings and other current work [12]. A key element of our concept is that the radiation oncologist at the non-particle radiotherapy institution finally decides who to treat locally and who to refer.

The concept of remote treatment plan comparison includes the necessity to send patient data out of a clinical institution and is implemented in ReCompare by using network connections as it was done, e.g., for the ROCOCO trial [13]. We took several measures to ensure data protection. First, only the non-particle radiotherapy institution has access to the full patient data since pseudonymization and depseudonymization happen locally. Second, all patient data are sent via a secure protocol (HTTPS) and the data transfer process is checked for completeness. Third, the server—being remotely accessible as well as protected by a restrictive firewall—is implemented to comply with data protection standards. Finally, the entire concept depends on the informed consent of the patient and we provide appropriate documentation and forms.

The presented findings encourage to apply the proposed concept and the software ReCompare on a larger clinical scale involving several radiotherapy institutions. A practical trial is currently initiated between the university hospital in Dresden as a particle therapy center and a number of non-particle radiotherapy institutions in Europe. Furthermore, the proposed concept may also support the comparison of photon plans optimized at different institutions (using different techniques). Presumably, a fully established expert system that supports the treatment decision by additionally including reliable state-of-the art clinical and radiobiological data and models will supersede the current approach [14]. Our work serves as a first step on the way to design and implement such an approach.

We believe that the benefits of our proposed concept are threefold. First of all, patients may benefit. Most of them are initially referred to a non-particle radiotherapy institution. Based on a treatment plan comparison the patient-specific allocation to a treatment modality may become more robust. Especially at remote institutions the chance for a referral of a patient with a high probability to profit from particle therapy may increase. Second, the non-particle radiotherapy institutions clearly benefit from the extra expertise regarding particle therapy which they get provided in the form of treatment plans. The continuous exchange should also serve as basis for the formation of networks between institutions with and without particle irradiation. And finally, particle therapy centers may benefit from an improved (pre-) selection of patients on an individual basis. Being able to exclusively treat those patients that are most profiting could maximize the efficacy of a particle irradiation (facility) and therefore justify the reimbursement of higher treatment expenses—usually associated with such a specialized treatment technique—by health insurances both in general and for individual patients.

## Conclusions

Our concept may help to establish a fair and sufficient access to specialized radiotherapy resources based on a standardized treatment plan comparison performed at non-particle radiotherapy institutions. The software tool ReCompare provides a secure and simple web-based exchange of photon and particle treatment plans. It will be available from our website when proton therapy is operational at our facility.

## Competing interests

The authors declare that they have no competing interests.

## Authors’ contributions

MB and WE: idea and concept. AL: drafting the manuscript and project coordinator. UJ, SH, SL, and AL: design of software. KR, SL, and AL: development of software. JP and AJ: testing of software. MK and AL: final revision of manuscript. All authors read and approved the manuscript.

## Authors’ information

Wolfgang Enghardt and Michael Baumann share last authorship.

## References

[B1] PTCOG Web pages http://ptcog.web.psi.ch/

[B2] Langendijk JA Lambin P de Ruysscher D Widder J Bos M Verheij M Selection of patients for radiotherapy with protons aiming at reduction of side effects: the model-based approach Radiother Oncol 2013 107 267 273 10.1016/j.radonc.2013.05.007 23759662

[B3] Grau C The model-based approach to clinical studies in particle radiotherapy – a new concept in evidence based radiation oncology? Radiother Oncol 2013 107 265 266 10.1016/j.radonc.2013.06.031 23890961

[B4] Marks LB Yorke ED Jackson A ten Haken RK Constine LS Eisbruch A Bentzen SM Nam J Deasy JO Use of normal tissue complication probability models in the clinic Int J Radiat Oncol 2010 76 S10 S19 10.1016/j.ijrobp.2009.07.1754 PMC4041542 20171502

[B5] ALLEGRO Group ALLEGRO Radiother Oncol 2012 105 6 144

[B6] Allen AM Pawlicki T Dong L Fourkal E Buyyounouski M Cengel K Plastaras J Bucci MK Yock TI Bonilla L Price R Harris EE Konski AA An evidence based review of proton beam therapy: the report of ASTRO’s emerging technology committee Radiother Oncol 2012 103 8 11 10.1016/j.radonc.2012.02.001 22405807

[B7] Gahbauer R Santiago A Grégoire V Begg A van der Kogel A Baumann M Enghardt W Bassler N Singers B D.JRA 3.2 – Report of different methods available for measurement of radiobiological relevant parameters in patients 2011 https://espace.cern.ch/ULICE-results/Shared%20Documents/D%20JRA_3%202_public.pdf

[B8] Roelofs E Engelsman M Rasch C Persoon L Qamhiyeh S de Ruysscher D Verhaegen F Pijls-Johannesma M Lambin P ROCOCO Consortium Results of a multicentric in silico clinical trial (ROCOCO): comparing radiotherapy with photons and protons for non-small cell lung cancer J Thorac Oncol Off Publ Int Assoc Study Lung Cancer 2012 7 165 176 10.1097/JTO.0b013e31823529fc 22071782

[B9] National Electrical Manufacturers Association Digital Imaging and Communications in Medicine (DICOM). Part 1: Introduction and Overview 2011 1300 N. 17th Street Rosslyn, Virginia 22209 USA: National Electrical Manufacturers Association

[B10] FIPS 197: advanced encryption standard http://csrc.nist.gov/publications/PubsFIPS.html

[B11] Ramaekers BLT Grutters JPC Pijls-Johannesma M Lambin P Joore MA Langendijk JA Protons in head-and-neck cancer: bridging the Gap of evidence Int J Radiat Oncol 2013 85 1282 1288 10.1016/j.ijrobp.2012.11.006 23273998

[B12] Stuschke M Kaiser A Abu-Jawad J Pöttgen C Levegrün S Farr J Re-irradiation of recurrent head and neck carcinomas: comparison of robust intensity modulated proton therapy treatment plans with helical tomotherapy Radiat Oncol Lond Engl 2013 8 93 10.1186/1748-717X-8-93 PMC3648492 23601204

[B13] Roelofs E Persoon L Qamhiyeh S Verhaegen F de Ruysscher D Scholz M Iancu G Engelsman M Rasch C Zijp L Meerleer GD Coghe M Langendijk J Schilstra C Pijls-Johannesma M Lambin P Design of and technical challenges involved in a framework for multicentric radiotherapy treatment planning studies Radiother Oncol 2010 97 567 571 10.1016/j.radonc.2010.08.009 20864198

[B14] Lambin P van Stiphout RGPM Starmans MHW Rios-Velazquez E Nalbantov G Aerts HJWL Roelofs E van Elmpt W Boutros PC Granone P Valentini V Begg AC de Ruysscher D Dekker A Predicting outcomes in radiation oncology—multifactorial decision support systems Nat Rev Clin Oncol 2013 10 27 40 23165123 10.1038/nrclinonc.2012.196 PMC4555846

